# Editorial: Methods in respiratory pharmacology 2023

**DOI:** 10.3389/fphar.2025.1527053

**Published:** 2025-01-14

**Authors:** Nadia Milad, Giulio Cabrini, Maria Cordina

**Affiliations:** ^1^ Department of Medicine, McMaster University, Hamilton, ON, Canada; ^2^ Department of Life Sciences and Biotechnology, University of Ferrara, Ferrara, Italy; ^3^ Department of Clinical Pharmacology and Therapeutics, Faculty of Medicine and Surgery, University of Malta, Msida, Malta

**Keywords:** respiratory disease, methodology, pharmacology, therapeutics, editorial

It is our great pleasure to introduce the Research Topic “*Methods in Respiratory Pharmacology 2023*,” featured in this Research Topic of Frontiers in Pharmacology. This Research Topic underscores innovative methodologies, reflecting advancements that shape our understanding and treatment of respiratory diseases.

Respiratory pharmacology has significantly transformed with the development of sophisticated *in vitro* models and novel therapeutic strategies. This Research Topic encapsulates these advancements, providing valuable insights into experimental models and potential therapeutic agents that hold promise for improving patient outcomes.

One of the key contributions to this Research Topic is the article “*3D Cell Culture Models in Research: Applications to Lung Cancer Pharmacology*.” Lung cancer remains one of the leading causes of cancer-related mortality worldwide, necessitating innovative research methodologies to improve treatment outcomes. The advent of 3D cell culture techniques has marked a significant advancement in lung cancer research, offering more physiologically relevant models compared to traditional 2D cultures. These 3D models, including spheroids, organoids, and engineered tissue models, play pivotal roles in enhancing our understanding of lung cancer biology, facilitating drug development, and advancing precision medicine. By more closely mirroring human lung tumors, these models pave the way for more effective and personalized therapeutic strategies (Vella et al.).

Another notable manuscript is “*A Novel In Vitro Cell Model of the Proteinase/Antiproteinase Balance Observed in Alpha-1 Antitrypsin Deficiency*.” Alpha-1 antitrypsin deficiency (AATD) is a genetic condition that disrupts the proteinase/antiproteinase balance, leading to emphysema. Predicting and assessing human responses to therapeutic candidates from preclinical animal studies have been challenging. This study developed a physiologically relevant *in vitro* model of the proteinase/antiproteinase balance and assessed its predictive power for pharmacological efficacy. The results support an inhibitor threshold above which the activity footprint generation appears resistant to increasing dosage, helping test inhibitors and assess NSP activity in emphysema (Chen et al.).

The issue also includes a significant clinical study titled “*The Use of Antibiotics in the Early Stage of Acute Exacerbation of Chronic Obstructive Pulmonary Disease (AECOPD) in Patients Without Obvious Signs of Infection: A Multicenter, Randomized, Parallel Controlled Study*.” The use of antibiotics in the early stage of AECOPD in patients without obvious signs of infection is a contentious issue. This multicenter, randomized, parallel controlled study sought to clarify whether this treatment method, in the absence of clear infections indicators, has an impact on short- and long-term prognosis of AECOPD patients. The study found that while overall rates of exacerbation frequency, ICU treatment, and mortality were not significantly different between the antibiotic and nonantibiotic groups, the 30-day readmission rate was lower in the antibiotic group. Additionally, patients in the antibiotic group had shorter hospital stays and lower CRP levels and sputum viscosity at the 30-day follow-up, suggesting that antibiotics may improve short-term outcomes in specific subsets of AECOPD patients (Zhou et al.).

Lastly, the article “*Vandetanib as a Prospective Anti-inflammatory and Anti-contractile Agent in Asthma*” explores the therapeutic potential of vandetanib, a tyrosine kinase inhibitor, in treating asthma. Vandetanib, a tyrosine kinase inhibitor, primarily exerts therapeutic effects in lung cancers, but its potential benefits in asthma were unclear. This study investigated vandetanib’s anticontractile and anti-inflammatory properties in asthma, where *in vivo* experiments in an asthmatic mouse model showed that vandetanib alleviates systemic inflammation and various airway pathological changes, including hypersensitivity, hypersecretion, and remodeling. *In vitro* experiments demonstrated that vandetanib relaxes precontracted tracheal rings via calcium mobilization regulated by specific ion channels. These findings suggest the feasibility of using vandetanib to reduce abnormal airway contraction and systemic inflammation in asthma (Zeng et al.).

The contributions to this Research Topic underscore the dynamic and interdisciplinary nature of respiratory pharmacology. They reflect ongoing efforts to develop more effective treatments for respiratory diseases through innovative research and collaborative endeavors. We extend our gratitude to all authors and reviewers for their invaluable contributions and insights.

As editors, we are excited to share these advancements with the scientific community and hope this Research Topic inspires further research and innovation in respiratory pharmacology. We invite you to explore these articles and engage with the groundbreaking work presented in this Research Topic.

